# Direct and opportunity costs related to utilizing maternity waiting homes in rural Zambia

**DOI:** 10.1016/j.midw.2021.103211

**Published:** 2022-02

**Authors:** HaEun Lee, Elisa M. Maffioli, Philip T. Veliz, Isaac Sakala, Nchimunya M. Chiboola, Jody R. Lori

**Affiliations:** a School of Nursing, University of Michigan, 400 N Ingalls St. Ann Arbor, MI 48104, United States; b School of Public Health, University of Michigan, 1415 Washington Heights, Ann Arbor, MI 48109, United States; c Africare Zambia, Flat A, Plot 2407/10 MBX, Off Twin Palm Road, Ibex Hill, Box 33921 Lusaka, Zambia

**Keywords:** Maternity waiting home, Mothers’ shelter, Opportunity cost, Direct cost, Financial barrier, Zambia

## Abstract

**Aim:**

To assess the direct and opportunity costs involved in utilising maternity waiting homes.

**Method:**

A cross-sectional admission survey administered to women who used ten maternity waiting homes across two rural districts in Zambia. A total of 3,796 women participated in the survey. Descriptive analysis was conducted on three domains of the data: demographic characteristics of women, direct costs, and opportunity costs.

**Findings:**

Waiting to deliver (86.3%), safe birth (70.8%), and distance (56.0%) were the most frequent reasons women reported for using a maternity waiting home. In terms of direct costs, roughly 65% of the women brought seven days or fewer days' worth of food to the maternity waiting homes, with salt, mealie meals, and vegetables being the most frequently brought items. Only 5.8% of the women spent money on transport. More than half of the women reported paying user fees that ranged from 1 to 5 or more kwacha (US$0.10- 0.52). In terms of opportunity costs, 52% of the women participated in some form of income generating activities (IGAs) when at home. Approximately 35% of the women reported they lost earned income (1 to 50 or more kwacha) by staying at a maternity waiting home.

**Conclusion:**

A large proportion of women paid for food and user fees to access a maternity waiting home, while a low number of women paid for transport. Even though it is difficult to assign monetary value to women's household chores, being away from these responsibilities and the potential loss of earned income appear to remain a cost to accessing maternity waiting homes. More research is needed to understand how to overcome these financial constraints and assist women in utilising a maternity waiting home.

## Highlights

• Lack of financial resources is a critical barrier to utilising Maternity Waiting Homes (MWHs) in low-income countries (LICs).
• Food and user fees are most frequent expenditures for women utilising MWHs in rural Zambia.
• Being away from various household chores, the loss of income generating activities (IGAs), may also be a financial constraint in utilising MWHs.

## Introduction

Financial barriers are some of the most prevalent impediments for women of low-income countries (LICs) who desire access to crucial reproductive health services (Scott et al., 2018; Sialubanje et al., 2015; Tancred et al., 2016). These costs are often tied to services such as various screenings, prescribed medication, facility-based delivery, and postnatal care. A systematic review regarding drivers and deterrents of facility-based delivery in sub-Saharan Africa found that women with high household wealth and high socioeconomic status were more likely to use facility-based delivery (Moyer and Mustafa, 2013). High fees have also been identified as barriers to access antenatal and postnatal care services (Sacks et al., 2017; Sibanda et al., al.,2018). Consequently, the low utilisation of key reproductive health services contributes to the high maternal mortality and morbidity that are fundamentally preventable in LICs (Sacks et al., 2017). To alleviate the financial burden on women of low socioeconomic status, many countries in sub-Saharan Africa have abolished user fees for maternal, neonatal, and child health services since the early 2000s (Masiye et al., 2010; McPake et al., 2013). User fees are fees paid to the health facility to utilise certain health services (Dzakpasu et al., 2014). However, while abolishing the user fees has generally led to higher utilisation, costs involved in transport, delivery supplies, and informal payments remain considerable barriers to equity in access (Atuoye et al., 2015; Dodzo and Mhloyi, 2017; Kananura et al., 2017).

User fees involved in utilising Maternity Waiting Homes (MWHs) can be substantial. MWHs are a community based, equity-orientated intervention that has been endorsed by the World Health Organization as one component of a comprehensive package to reduce maternal morbidity and mortality (Bergen et al., 2019; Gabrysch et al., 2011). MWHs are lodgings located near health facilities where women can await labour and birth (Scott et al., 2018). The primary goal of MWHs is to improve maternal and infant outcomes for women living far from health facilities (Perosky et al., 2019). While there are no standard operating procedures or policies used across MWHs, general evidence suggests that women who have access to a health facility with a MWH in close proximity are more likely to receive professional healthcare before, during, and after their birth than women who have access to health facilities without a MWH (Chiu et al., 2019; Sialubanje et al., 2017). MWHs can be accessed by all women within the community but women at high obstetric risk are often referred by midwives and healthcare providers (Lori et al., 2018).

Similar to other low-income countries, studies from sub-Saharan African countries including Eritrea, Zimbabwe, Kenya, and Zambia show that the cost of using MWHs is a barrier to access for many women (Andemichael et al., 2009; Chibuye et al., 2018; Chiu et al., 2019; Mutea et al., 2015; Sialubanje et al., 2015; Tancred et al., 2016). The lack of financial resources for food, transport, baby clothes, and user fees are frequently and consistently identified as deterrents to women’s use of MWHs (Kyokan et al., 2016; Sialubanje et al., 2015). Furthermore, when women are not able to prepare birth items such as cotton gauze, plastic covers used in delivery, gloves, and clean clothes for the babies and themselves, they were less likely to utilise a MWH (Scott et al., 2018; Tancred et al., 2016; van Lonkhuijen et al., 2003).

In Zambia, a landlocked country in sub-Saharan Africa, MWHs have been integrated into the health system with formal linkages to healthcare facilities (Chibuye et al., 2018). As in other LICs, informal fees involved in MWH utilisation remain a potential barrier for women. While items such as food and user fees have been identified as significant barriers in Zambia, to the best of our knowledge, no study has examined the direct costs, nor the opportunity costs involved in utilising MWHs. Therefore, the purpose of this study was to gain better insight of direct costs and opportunity costs involved in accessing MWHs in rural Zambia.

## Methods

A secondary analysis was conducted on cross-sectional admission surveys collected from women staying at ten MWHs in the Mansa, Chembe, and Lundazi districts in Zambia between 2016 and 2018. Because MWHs have existed in Zambia for decades with generally low quality and no standardised policy, the primary aim of the parent study was to implement MWHs using a MWH core model with specific standards and policy in relation to understanding the impact of standardised MWHs on reproductive health service access (Scott et al., 2018). Ten MWHs were implemented in a quasi-experimental parent study with the aim of evaluating the impact of the introduction of MWHs on reproductive health service access and maternal health outcomes. To collect robust data for decision-makers on the effectiveness of MWHs in Zambia, the parent study developed a core model for MWHs in rural Zambia with criteria in three domains: 1) infrastructure, equipment, and supplies, 2) policies, management and finances, and 3) linkages and services (Lori et al., 2018). In collaboration with the Ministry of Health, the ten sites in the Mansa, Chembe, and Lundazi districts were identified for the MWHs to be implemented. The detailed process of choosing the implementation site for the MWH and further details regarding the parent study are reported elsewhere (Lori et al., 2016; Scott et al., 2018).

### Data collection

Data were collected daily via face-to-face interviews with women newly admitted to the MWHs between June 2016 and August 2018. At initial admission, the women were consented by local MWH caretakers, who are fluent in local languages. Women were informed they would be allowed to stay at the MWH regardless of their decision to participate in the interview. If the consent form was signed, the MWH caretakers proceeded with the interview by reading each of the questions in the survey and recording the answers. The surveys were then transcribed by the local research assistants into an Excel spreadsheet on a weekly basis. The detailed description of the collections tools development process is described elsewhere (Scott et al., 2018). Ethical approval was obtained from the Institutional Review Board (IRB) and the ERES Converge Research Ethics Committee of the authors’ institutions.

### Study setting

Zambia consists of 10 provinces with 74 districts and a total population of 17.09 million (World Bank, 2019). It suffers from a high poverty rate: 58% of the population live below the international poverty line of US $1.9 per day (World Bank, 2018). The fertility rate is 4.7 births per woman, whereas women living in rural areas have two more children on average as compared to those living in urban areas (Central Statistical Office et al., 2018). The majority of births (80%) are assisted by skilled health care professionals; however, there is a difference between urban (93%) and rural (79%) areas (Central Statistical Office et al., 2018).

Data were collected from women utilising ten MWHs in three districts, Mansa, Chembe, and Lundazi. The three districts were part of the Saving Mothers Giving Life (SMGL) initiative from 2012 to 2016 to reduce maternal and newborn mortality. This 5-year initiative was designed and implemented within the Global Health Initiative as a public-private partnership between the U.S government, Merck for Mothers, Every Mother Counts, the American College of Obstetricians and Gynecologists, the Norway government, and Project CURE (Kruck et al., 2016). Mansa has a population of 228,392 with 61.9% of the population living in rural areas and Lundazi a population of 323,870 with 95.1% of its population in rural areas (Central Statistical Office, 2010).

### Measures

The survey consisted of three separate domains: demographic characteristics, direct costs, and opportunity costs. The first domain contains of demographic questions that included age, gravida, parity, education level, number of companions (people who accompanied the women to MWH), types of companions (e.g. spouse/partner, mother, sister), and reasons for utilising the MWH. Women were allowed to choose more than one option for types of companion and the reason for coming to the MWH.

The second domain collected information on both monetary and non-monetary direct costs involved in utilising MWHs. These questions include the amount of money spent on food, transport, user fees, and the woman’s willingness to return if they were required to pay user fees. Furthermore, questions such as “what food item did you bring from home?” and “how many days of food did you bring from home?” were asked. For the items of food, women were allowed to choose more than one option provided. Direct cost of a specific illness or disease is often defined as the cost involved in both in-patient and out- patient services, such as visits to healthcare professionals as well as other expenses associated with diagnosis and treatment (Anandarajah et al., 2016; Panopalis and Clarke, 2006; Slawksy et al., 2011). However, because reproductive health services have been provided for free in Zambia since 2006 and because we are concerned with the costs involved in MWH utilisation, we included informal costs for food, transport, and user fees under the category of direct cost.

The last domain collected information about opportunity costs. This domain asked questions about the types of income generating activities (IGAs) the woman participated in, the type of activity in which the woman would be participating were she not at the MWH, and the amount of income she was losing by staying at the MWH (Keya et al., 2018). Opportunity costs were defined as the loss of potential gain from other alternatives when one alternative is chosen, such as the loss of income or opportunity due to inability to carry out specific activities (Anandarajah et al., 2016). It is much more challenging to accurately and comprehensively identify and calculate opportunity costs. Therefore, in this paper, we present two numerical figures for opportunity cost, one self-reported by the women and another calculated by multiplying the average monthly income per capita for rural households and the average number of days women stayed at the MWHs. Descriptive analysis was also conducted on the three domains of the data (demographic characteristics, direct costs, opportunity cost).

### Data analysis

The data were analysed using Stata 15.0 (StataCorp, College Station, TX, USA). The aim of this analysis was to 1) provide descriptive statistics for the demographic characteristics of the women, and 2) examine the direct costs and opportunity costs involved in utilising MWHs. Means and standard deviations (SD) were calculated to estimate the cost of food, transport, user fees, and lost income. Both conditional means, the average of those who paid anything for the specific category, and unconditional means, the average of those who paid anything and nothing for the specific category, were calculated. Frequency with percentages was calculated for categorical variables.

## Findings

Demographic characteristics of the women who participated in the survey are shown in Table 1. A total of 3796 women participated in the survey between June 2016 and August 2018. The majority of the women were between the ages of 16 and 25 years, with a mean of 24 (SD 6.3) years old. Women reported an average number of pregnancies of three and live births of two. Close to 70% of the women had some primary education. On average, women had two companions who accompanied them to the MWH. Most frequently this was a woman’s mother-in-law (57.5%) and/or mother/auntie (28.9%). Approximately 7.3% of the women came to the MWH alone and less than 1% of the women were accompanied by their husband. amongst the reasons for utilising the MWH, “waiting to birth” (86.3%) was the most frequently identified. This was followed in frequency by “safe birth” (70.8%) and “distance” (56.0%).

**Table 1:** Demographic characteristics of women.

**Characteristics**	

**Total, n**	3796
**Age (mean, SD)**	24.15 (6.30)
≤ 15, n (%)	22 (0.58)
16–20, n (%)	1351 (35.59)
21–25, n (%)	1106 (29.14)
26–30, n (%)	594 (15.65)
31–35, n (%)	418 (11.01)
36–40, n (%)	213 (5.61)
≥ 40, n (%)	41 (1.08)
Missing	51 (1.32)
**Gravida (mean, SD)**	3.06 (1.95)
Missing, n (%)	21 (0.55)
**Parity (mean, SD)**	1.94 (1.85)
Missing, n (%)	22 (0.58)
**Education, n (%)**	
No education	408(10.75)
Primary	2605(68.62)
Secondary	751 (19.78)
Missing	32 (0.84)
**Number of companions (mean, SD)**	1.88 (2.94)
Missing, n (%)	208 (5.48)
**Companion, n (%)**	
Mother in law	2185 (57.56)
Mother/ Auntie	1098 (28.93)
Husband	36 (0.95)
Sister	258 (6.80)
Friend	19 (0.50)
Traditional Birth Attendant	9 (0.24)
SMAG	74 (1.95)
Alone	278 (7.32)
Children	8 (0.21)
**Reason coming to MWH, n (%)**	
ANC problem/ illness	177 (4.66)
Waiting to deliver	3277 (86.33)
Stayed at MWH before	204 (5.37)
Comfort	1016 (26.77)
Safe delivery	2688 (70.81)
Distance	2129 (56.09)
Referred	319 (8.40)
Postpartum stay	351 (9.25)
Sick visit for recently delivered mother or baby	45 (1.19)

The direct costs involved in utilising MWHs are shown in Table 2**.** About 76.8% of the women who utilised MWH and have participated in the survey spent money on food. Close to 45% of the women brought fewer than 7 days’ worth of food and 20% of the women brought a week’s worth of food. Mealie meal (75.6%), salt (64.4%), and vegetables (64.1%) were some of the most commonly brought food items. On average, women who reported spending any money on food spent 32 kwacha ($3.35). The average conversion rate for 2017 was used to convert Zambian Kwacha to US dollars. When combined with those who did not spend any money on food, women on average spent 28.5 kwacha ($2.98). Approximately 44% of the women spent less than 20 kwacha ($2.09) on food and approximately 18% of the women spent 30–50 kwacha. A majority of the women did not spend any money on transport (75.3%); however, those who did spent approximately 12 kwacha ($1.25) on average. When combined with those who did not spend any money on transport, women spent about 1.8 kwacha ($0.18) on average. The distance travelled by the women ranged from less than a kilometre to more than 10 kms (Lori et al., 2021). Detailed results related to distance and types of transport are reported elsewhere (Lori et al., 2021). Women spent different amounts of money on user fees, ranging from none to 5 or more kwacha, upon admission depending on the policy of each MWH. Approximately 20% of the women did not pay a user fee to stay at the MWH; however, those who did paid 2.5 kwacha ($0.25). When the average was taken from both groups of women who paid and did not pay user fees, women paid 1.8 kwacha ($0.18) on average. A majority of the women (77.5%) also responded they would return even if they were required to pay user fee. Overall, when all the average costs of food, transport and user fees are combined for all the women in the survey (unconditional means), women who utilised MWHs spent about 31.83 kwacha ($ 3.33) in total. The direct costs involved in MWH utilisation was 46.5 kwacha ($4.86) on the sample of women who spent any positive amount on food, transport, and user fees (conditional means).

**Table 2:** Direct costs.

**Characteristics**	

**Days of food brought to MWH, n (%)**	
None	86 (2.18)
< 7 days	1647 (43.38)
7 days	755 (19.88)
8–14 days	431 (11,35)
>15 days	84 (2.21)
Missing	793 (20.89)
**Items of food brought, n (%)**	
Sugar	1265 (33.32)
Salt	2447 (64.46)
Mealie meal	2, 871 (75.63)
Vegetable	2436 (64.17)
Fruit	435 (11.46)
Cooking oil	1573 (41.44)
Rice	325 (8.56)
Fish	576 (15.17)
Chicken	144 (3.79)
Other meat	173 (4.56)
Drink	194 (5.11)
Other food	470 (12.38)
Wood	1137 (29.95)
Charcoal	494 (13.01)
**Amount of money spent on food**	
⁎Conditional a (mean, SD)	32.03 (40.0)
⁎⁎Unconditional a (mean, SD)	28.57 (39.1)
None, n (%)	280 (7.38)
1–20 a	1669 (43.97)
30–50 a	674 (17.76)
>50 a	392 (8.67)
Missing	843 (22.23)
**Paid for transportation**	
⁎Conditional a (mean, SD)	12.00 (20.6)
⁎⁎Unconditional a (mean, SD)	1.43 (8.0)
No, n (%)	2860 (75.34)
Yes, n (%)	220 (5.80)
1–10 a	174 (4.58)
>10 a	48 (1.26)
Missing	716 (18.86)
**Paid to stay at MWH**	
⁎Conditional a (mean, SD)	2.49 (0.7)
⁎⁎Unconditional a (mean, SD)	1.83 (1.2)
No, n (%)	793 (20.89)
Yes, n (%)	
1–2	1310 (34.51)
3	804 (21.18)
5 or more	96 (2.53)
Missing	793 (20.89)
**Comeback if have to pay, n (%)**	
No	38 (1.00)
Yes	2942 (77.50)
Missing	811 (21.36)

a expressed in Zambian kwacha: 2017 Conversion rate 1kwacha= $0.104.

⁎ the average of those who paid anything for the specific category.

⁎⁎ the average of those who paid anything and nothing for the specific category.

Finally, in Table 3 the opportunity costs lost over the days spent at the MWH are shown. A little over half of the women (52.2%) reported engaging in some form of Income Generating Activities (IGAs). The types of activities in which women would have been participating if not at the MWH included a list of both income and non-IGAs. Farming (87.1%) was most common, followed by caring for a husband (41.0%), caring for children (36.9%), and/or taking care of the house (36.7%), and gathering water (33.6%). When women were asked to self-report if they would lose any income by staying at the MWH, 64.8% of women answered no and 35.1% of women answered yes. Questions regarding cost of food and food items brought were added later to the pre-existing survey. As such, the women who took part in the survey in the beginning did not answer these questions. The table reflects the percentages of missing answers. The estimated lost income ranged from 1 to 50 or more kwacha, with the average lost income reported by the women being 30.7 kwacha ($3.21). When combined with all women who answered the question either yes or no, the average lost income was 10.2 kwacha ($1.06). However, when the opportunity cost was calculated based on the monthly income of 185 kwacha per capital for rural households and the average number of days women stayed at the MWHs (16 days), the potential financial cost from loss of work productivity was 98.6 kwacha ($10.32) (Central Statistical Office, 2016).

**Table 3:** Opportunity costs.

**Characteristics**	

**IGA**^a^**participation, n (%)**	
No	1760 (46.36)
Yes	1982 (52.21)
Missing	54 (1.42)
**Type of activity, n (%)**	
Farming	3307 (87.12)
Caring for children	1403 (36.96)
Caring for parents	1038 (27.34)
Caring for husband	1558 (41.04)
Gathering water	1279 (33.69)
Caring for animals	590 (15.54)
Laundry	950 (25.03)
Caring for house	1394 (36.72)
Food processing	409 (10.77)
Selling at market	96 (2.53)
Community work	639 (16.83)
Piece work	161 (4.24)
**Lost income**	
⁎Conditional b(mean, SD)	30.79 (45.8)
⁎⁎Unconditional b (mean, SD)	10.21 (30.1)
Calculated based on days stayed at the MWH b	98.66
No, n (%)	2463 (64.88)
Yes, n (%)	1333 (35.11)
1–10 b	377 (9.93)
11–20 b	392 (10.33)
21–30 b	95 (2.50)
31–50 b	240 (6.32)
>50 b	119 (3.13)
Missing	110 (2.90)

a IGA: income generating activity.

b expressed in Zambian kwacha: 2017 Conversion rate 1kwacha= $0.104.

⁎ the average of those who paid anything for the specific category.

⁎⁎ the average of those who paid anything and nothing for the specific category.

## Discussion

We examined the direct costs and opportunity costs of utilising a MWH in rural Zambia. In our study we found that a large proportion of the women who utilised MWHs not only carried various food items for anticipated long-term stays at the MWHs, but also spent money on food once at the MWH. Carrying food amongst other items can be a barrier to utilising MWHs given the knowledge that the MWHs specifically target women living in rural areas, far away from health facilities. The MWHs implemented by the parent study provided basic food such as mealie meal to pregnant women. However, the researchers found a majority of the women still felt the need to carry additional food. This finding aligned with other studies where women feared there would be insufficient food for themselves and their companions and described food insecurity as a major barrier to utilising MWHs (Chibuye et al., 2018; Lori et al., 2016; Sialubanje et al., 2015). Furthermore, women purchased more food even after bringing several days’ worth of food with them to the MWHs.

Varying operational models for MWHs exist in Zambia, and each have different policies regarding food. Some only offer food to the pregnant women, while others provide food to their companions as well (Chibuye et al., 2018). However, most MWHs allow women to cook for themselves in separate kitchens or outdoor facilities associated with the MWH (Chibuye et al., 2018). More studies are needed to examine the cost of food while staying at the MWHs.

Previous studies have also identified transport costs as a barrier to accessing MWHs (Kyokan et al., 2016; Scott et al., 2018). However, our findings suggest that the majority of women do not spend money on transport, suggesting the cost of transport does not appear to be a major barrier in our setting. The MWHs specifically target the delay in reaching care. They allow women to come and await birth rather than having them travel once the labour has started. When a woman tries to reach a health facility following the onset of labour, finding and paying for transport is a priority and a much bigger barrier than during pregnancy. Therefore, it is possible that, while the cost of transport is not a financial barrier to access MWHs in Zambia, it remains a potential limit during labour. More research is needed to understand why transport is not a substantial cost in accessing MWHs in this context.

We also found a large number of women paid user fees and were willing to return despite user fees. However, the literature is more mixed regarding user fees. Some studies suggest women are willing to financially contribute to their stay at a MWH, while others suggest that user fees will impede access, especially for the poor (Borghi et al., 2006; Sibanda et al., 2018). A qualitative study based on the gathered perspectives of pregnant women, mothers, health care workers, and community members showed that participants at MWHs express a concern that women will be deterred by user fees because other reproductive health services have been free in Zambia since 2006 (Chibuye et al., 2018). The literature on MWH user fees is scant, and more studies need to be conducted to understand the appropriate cost of fees and their effect on use.

There was a large discrepancy between the estimated opportunity cost that was self-reported by the women and the estimation based on the average number of days women stayed at the MWH and the average monthly income in rural Zambia. While a little over half of the women participated in some form of IGAs, only over a third of the women reported that they lost income while staying at the MWH. There may be many reasons contributing to the discrepancy between the two types of opportunity cost estimation. The estimated opportunity cost based on the average number of days women stayed at the MWH and the average monthly income in rural Zambia provides a more unbiased estimation given that respondents may have been hesitant to share their true loss of income. However, the value should be interpreted cautiously since the average monthly income in rural Zambia used to compute the opportunity cost is the national average. Since there is potential variation in the type of jobs and household chores women are involved in, the monthly income may vary amongst women in our sample. This once again emphasises the difficulty in estimating opportunity cost because women’s labour is less easily translated monetarily compared to men. Even though the labour burden of rural Zambian women exceeds that of men, opportunity costs include a high proportion of unpaid household responsibilities such as caring for children, preparing food, and collecting fuel and water. Leaving these responsibilities can be a crucial barrier in utilising MWHs.

If not at the MWH, some of the women would be participating in IGAs including farming, food processing, and selling at markets. Farming was most often identified as a rural Zambian women’s daily activity compared to all IGAs and non-IGAs. However, despite women constituting 64% of Zambia’s rural population and approximately 80% of food producers, a majority of women in agriculture (59.3%) are unpaid family workers, meaning they often work on their own or on their family’s farm without pay (Food and Agricultural Organization of the United Nation, 2018). Furthermore, our study found that a low percentage of women was selling at markets. This finding aligns with a recent report that shows women’s participation at wholesale markets remains limited due to an unfriendly environment for women within markets (Food and Agricultural Organization of the United Nations, 2018).

This study has several limitations. First, because the survey was conducted only with women who utilised a MWH despite the constraints, there is a sample bias. Women who faced the greatest financial barriers and were not able to access a MWH were not captured in the survey. Furthermore, because the data were only collected from the ten MWHs implemented by the parent study, women who utilised MWHs that are under different operational models were not captured. Second, because survey answers were reported through the MWH caretaker, there is a risk of social desirability bias. The women may have over reported the positive aspects of utilising the MWHs, especially when asked about their reasons for utilising the MWHs or their willingness to return to the MWH despite user fees. However, due to women’s limited literacy in rural Zambia, it was necessary to go through the MWH caretakers. It was clearly explained before collecting the consent form and further data from the women that they would still be able to use the MWH and there would be no disadvantages if they declined the interview. Third, the questions regarding cost of food and food items brought were added to the survey. This resulted in not capturing data from the women who took part in the survey at the start of the study and did not answer these questions. However, the number of missing answers is not substantial, and the tables reflect the percentages of missing answers. Lastly, the survey did not collect all cost figures, so it is difficult to calculate the precise value of direct and opportunity costs. Despite these limitations, the present study provides important insights regarding direct and opportunity costs involved in utilising the MWHs.

## Conclusion

To the best of our knowledge, this study is the first to examine the direct and opportunity costs involved in utilising a MWH in rural Zambia. Despite MWHs having great potential to improve maternal and child health outcomes, the lack of financial resources is a critical barrier to utilising them. The present study shows that a large proportion of women who utilised MWHs in our study also pay for food and user fees, while a low number of women paid for transport. amongst different categories of direct costs, food was the most substantial expense. The study also found that by being away from various household chores the loss of IGAs may also be a financial constraint in utilising MWHs due to multiple responsibilities that women hold. More research is needed to understand the various costs involved in utilising MWHs and how to overcome these financial constraints to assist women in utilising MWHs.

## Funding sources

This work was implemented in collaboration with *Merck for Mothers*, Merck’s 10-year, $500 million initiative to help create a world where no woman dies giving life. *Merck for Mothers* is known as *MSD for Mothers* outside the United States and Canada (MRK 1846–06,500.COL). The development of this article was additionally supported in part by the 10.13039/100000865Bill & Melinda Gates Foundation (OPP1130334) https://www.gatesfoundation.org/How-We-Work/Quick-Links/Grants-Database/Grants/2015/06/OPP1130334 and The ELMA Foundation (ELMA-15-F0010) http://www.elmaphilanthropies.org/the-elma-foundation/. The funders had no role in study design, data collection and analysis, decision to publish, or preparation of the manuscript. The content is solely the responsibility of the authors and does not necessarily reflect positions or policies of Merck, the Bill & Melinda Gates Foundation, or the ELMA Foundation.

## Declaration of Competing Interest

The authors declare they have no competing interests.
